# Correlation between percutaneous patent foramen ovale closure and recurrence of unexplained syncope

**DOI:** 10.3389/fneur.2023.1104621

**Published:** 2023-02-01

**Authors:** Xianwen Wang, Xiangwei Liu, Lulu Zheng, Yubo Liu, Zhengyan Guan, Jingyi Dai, Xiaobin Chen

**Affiliations:** ^1^Department of Cardiology, Xiangya Hospital, Central South University, Changsha, Hunan, China; ^2^Department of Cardiology, The Eighth Affiliated Hospital, Sun Yat-sen University, Shenzhen, Guangdong, China

**Keywords:** patent foramen ovale, unexplained syncope, right-to-left shunt, paradoxical embolism, residual shunt

## Abstract

**Background:**

The relationship between patent foramen ovale (PFO) and unexplained syncope remains to be illustrated. Therefore, this study aimed to explore the outcomes and prognostic factors for syncope recurrence after PFO closure.

**Methods:**

Patients with both large right-to-left shunting (RLS) PFO and unexplained syncope who visited the cardiovascular department of Xiangya Hospital Central South University from 1 January 2017 to 31 December 2021 were consecutively enrolled in our study. The recurrence rate of syncope was compared between the non-closure group (*n* = 20) and the closure group (*n* = 91).

**Results:**

A total of 111 patients were finally included. After 31.11 ± 14.30 months of follow-up, only 11% of patients in the closure group had recurrent syncope, which was much lower than that of the non-closure group (11.0 vs. 35%, *P* = 0.018). We further investigated the possible prognostic factors for syncope recurrence in the closure group and found syncope occurring more than five times preoperatively, hypertension, and residual RLS at 12-month follow-up were significantly correlated with a higher number of recurrences.

**Conclusions:**

PFO closure reduced the recurrence rate of unexplained syncope. The efficacy of prevention was prognosticated by factors including the presence or absence of syncope induction, the frequency of syncope episodes, and the presence or absence of hypertension. Syncope recurrence was also related to residual shunts post closure.

## 1. Introduction

The patent foramen ovale (PFO), a remnant of the fetal foramen ovale, is a risk factor for cryptogenic stroke (1, 2). According to relevant statistics, the incidence of PFO in adults is ~25% (3–5). With the release of long-term follow-up results of four major clinical trials (6–9), many countries and regions have updated the guidelines related to PFO closure (10–14). The American Academy of Neurology (AAN) guideline (15) suggests that, in patients with a PFO and no other etiology for stroke, PFO closure probably reduces recurrent stroke risk in select patients younger than 60 years with an embolic-appearing stroke. In addition, the Society for Cardiovascular Angiography and Interventions (SCAI) guidelines (16) recommend PFO closure rather than antiplatelet therapy alone in patients between the ages of 18 and 60 years with a prior PFO-associated stroke [PFO-AS (17)]. For patients without PFO-AS, the SCAI guideline panel advises against the routine use of PFO closure, except in the following special cases. Patients, particularly those with debilitating migraines who have failed to benefit from conventional medical therapy or those with decompression illness who have a strong desire, may reasonably choose PFO closure. In persons with platypnea-orthodeoxia syndrome (POS), in whom other causes of hypoxia have been excluded, the panel recommends PFO closure. In persons with systemic embolism, in whom other embolic etiologies have been excluded, the panel suggests PFO closure rather than medical therapy alone. The association of PFO with neurological diseases caused by a paradoxical embolism (ischemic stroke, migraine, etc.) has been widely recognized. Syncope was also mentioned in these recommendations; however, the evidence levels were still not strong enough.

Syncope is a transient loss of consciousness caused by transient cerebral hypoperfusion and a fall due to decreased muscle tone and the inability to maintain a normal position (18). Unexplained syncope refers to a group of clinical syndromes in which the organic cause of the disease has not been identified after a detailed history, physical examination, and auxiliary examinations (19). In clinical practice, we often encounter patients with unexplained syncope accompanied by PFO, and many patients do not have a recurrence of syncope after PFO closure. A large single-center study showed that, for patients with cryptogenic cerebral ischemia (stroke/TIA/MRI ischemic lesion), PFO closure not only reduces the recurrence risk of cerebral ischemic events but also significantly decreases the incidences of migraine headache, platypnea-orthodeoxia, fainting episodes, syncope, and coenesthesia phenomena (20). However, there are few studies on the relationship between PFO and unexplained syncope. Therefore, the aim of our study was to explore the relationship between PFO closure and the recurrence of unexplained syncope as well as prognostic factors.

## 2. Materials and methods

### 2.1. Patients

A total of 118 patients with unexplained syncope and large right-to-left shunting (RLS) PFO who were admitted to the Department of Cardiovascular Medicine, Xiangya Hospital, Central South University, between January 2017 and December 2021 were included continuously. A total of 111 patients were finally screened according to the following inclusion and exclusion criteria and analyzed retrospectively. In patients with a large shunting PFO and unexplained syncope, we would recommend PFO closure if they had a PFO-associated stroke, migraine, decompression illness, POS, or systemic embolism. However, if the patients do not have PFO-associated diseases, we recommend against PFO closure unless there is a strong desire. The uncertain benefits and possible risks of PFO closure would be explained to the patients before the procedure, and PFO closure would be performed after the patients' consent. There were only 20 patients who did not undergo PFO closure because of their choice, and they were used as the control group. The other 91 patients who underwent PFO closure were used as the closure group.

The inclusion criteria were as follows: (1) Patients diagnosed with syncope according to the diagnostic criteria for syncope in the *2018 ESC Guidelines for the diagnosis and management of syncope* (21) and (2) a patent foramen ovale with a large RLS (evaluation methods are described in part 2.2).

The exclusion criteria were as follows: (1) CT or magnetic resonance imaging of the brain revealed lesions associated with syncope. Definite PFO-associated lesions that expert neurologists evaluated were not included; (2) Holter examination indicating arrhythmias with risk of cardiogenic syncope; (3) color Doppler ultrasound of neck vessels indicating cervical vascular lesions that could lead to syncope; (4) presence of infectious diseases, autoimmune diseases, blood system-related diseases; (5) presence of other cardiovascular abnormalities, other neurological disorders, and contraindications to PFO closure (such as pulmonary arterial hypertension); (6) patients younger than 12 years or older than 65 years of age; (7) unavailable complete clinical data, test results, and follow-up data.

The study was conducted in accordance with the Declaration of Helsinki and approved by the Research Ethics Committee of Xiangya Hospital (protocol code: 202209198). Informed consent was obtained from all subjects involved in the study.

### 2.2. Identification of the patent foramen ovale

PFO was identified using a multimodality screening method that included contrast transcranial Doppler ultrasonography (cTCD), transthoracic echocardiography (TTE), and contrast transthoracic echocardiography (cTTE). Transesophageal echocardiography (TEE) was performed in patients with atrial septal tumors (ASAs) or atrial septal defects (ASDs). According to the number of microbubbles in the left atrium at the resting state and after Valsalva action, the RLS of PFO was classified into four grades (22): (1) grade 0, no shunt (no microbubble); (2) grade 1, small shunt (1–10 microbubbles); (3) grade 2, middle shunt (11–30 microbubbles); and (4) grade 3, large shunt (>30 microbubbles or full of microbubbles in the left atrium).

### 2.3. Percutaneous patent foramen ovale closure

The same experienced interventional cardiologist performed the procedure in the cardiac catheterization room. The patients received oral aspirin of 3–5 mg/kg one time a day and clopidogrel of 50–75 mg one time a day 48 h before the operation. After local anesthesia, access to the patient's femoral vein was established, and the delivery sheath tube was delivered to the left superior pulmonary vein through the unclosed foramen ovale. The appropriate type of Cari-O-Fix PFO occluder or atrial septal defect occluder (ASDO) was selected according to the preoperative color Doppler ultrasound results. After careful examination by bedside color ultrasound and x-ray fluoroscopy and a parallel push-pull test, followed by a demonstration that the occluder was suitable in size, in a good position, and fixed, without the presence of a visible residual shunt and without affecting the function of the heart valve, the occluder was completely released. Heparin (100 U/kg) was intravenously injected during the operation, and low molecular weight heparin (100 U/kg) was subcutaneously injected Q12 h on the first day after the operation to prevent embolism. Additionally, aspirin (100 mg/day) and clopidogrel (75 mg/day) were taken orally for 6 months.

### 2.4. Outcome and follow-up

In the current study, the main clinical outcome was syncope recurrence. Demographic data (sex, age), clinical characteristics (hypertension, diabetes, and PFO-related diseases/symptoms), and details of all cases of syncope (historical time of syncope, duration of syncope (minutes), number of syncope episodes, and syncope triggers) were collected. Syncope triggers include exercise, defecation, urination, postural changes, emotional agitation, coughing, and lifting heavy objects. Routine c-TTE/TTE and electrocardiography were performed 1, 6, and 12 months after closure. A TEE was performed when necessary. These tests were used to assess residual shunt, effective closure rate [effective closure was defined as no shunt or a small shunt as indicated by postoperative c-TTE (8)], and arrhythmia after the operation. The recurrence of syncope, postoperative surgical complications, and other health conditions were investigated by telephone contact and a review of outpatient follow-up records.

### 2.5. Statistical analysis

All statistical analyses were performed using the Statistical Package for IBM SPSS Statistics for Windows (Version 26.0, Chicago, IL, USA). Means and standard deviations were calculated for parametric variables, and the medians and quartiles were calculated for non-parametric variables. The quantitative variables between the two groups were compared using two-sample *t*-tests (parametric) and the Mann–Whitney U tests (non-parametric). The dichotomous variables between the two groups were compared using Pearson's χ2 or Fisher's exact test, as appropriate. The rank variables between the two groups were compared using the Mann–Whitney U tests. Univariate and multivariable logistic regression models were used to analyze the prognostic factors for syncope recurrence after PFO closure. A probability (*p*) of < 0.05 was considered statistically significant.

## 3. Results

A total of 111 patients [68 (61.3%) women and 43 (38.7%) men] were included in this study; the average age of the patients was 40.41 ± 15.23 years. The baseline demographics and clinical features are shown in Table 1. The PFO closure group was more likely to have syncope triggers (53.8 vs. 25.0%) and headache symptoms (31.9 vs. 5.0%) but less likely to have a syncope episode (20.9 vs. 45.0%) or syncope symptoms only (without cerebral infarction, dizziness, headache, or epilepsy) (39.6 vs. 65.0%) than the control group. Among the 111 patients we enrolled, 54 (48.6%) identified syncope triggers, which included exercise (23 patients), defecation (7 patients), urination (6 patients), postural changes (5 patients), emotional agitation (4 patients), coughing (2 patients), lifting heavy objects (1 patient), and other (6 patients). There were no significant differences in sex, age, history of syncope, seizure time, seizure frequency, PFO- associated brain lesions, dizziness, epilepsy, or baseline ultrasound characteristics between the two groups.

**Table 1:** Comparisons of participant characteristics between the closure group and the control group.

	**Control group** **(*n* = 20)**	**Closure group** **(*n* = 91)**	***P*-value**
Sex: Women	11 (55.0)	57 (62.6)	0.526
Age (years)	39.25 ± 14.27	40.66 ± 15.50	0.504
Time of syncope history			0.107
Several days	6 (30.0)	10 (11.0)	
Several months	5 (25.0)	27 (29.7)	
Several years	9 (45)	54 (59.3)	
Duration of syncope onset			0.885
Several seconds	6 (30.0)	24 (26.4)	
Several minutes	11 (55.0)	55 (60.4)	
≥10 min	3 (15.0)	12 (13.2)	
Number of syncope episodes			0.103
1 time	9 (45.0)	21 (23.1)	
2–5 times	7 (35.0)	45 (49.5)	
>5 times	4 (20.0)	25 (27.5)	
First syncope episode	9 (45.0)	19 (20.9)	0.025
Syncope triggers	5 (25.0)	49 (53.8)	0.019
Syncope symptoms alone	13 (65.0)	36 (39.6)	0.038
PFO-associated brain lesions	2 (10.0)	7 (7.7)	1.000
Migraine	1 (5.0)	29 (31.9)	0.014
Dizziness	3 (15.0)	21 (23.1)	0.621
Epilepsy	2 (10.0)	4 (4.4)	0.647
Hypertension	3(15.0)	8 (8.8)	0.669
Diabetes mellitus	1 (5.0)	2 (2.2)	0.452
Size of atrial thin zone (mm)	18.80 ± 3.40	17.75 ± 4.71	0.149
Preoperative resting RLS grade			0.274
No	9 (45.0)	56 (61.5)	
Small	6 (30.0)	18 (19.8)	
Middle	3 (15.0)	3 (3.3)	
Large	2 (10.0)	14 (15.4)	
Preoperative RLS grade			
Large	20 (100.0)	64 (100.0)	1.000
Preoperative right echocardiography			0.413
Negative	0 (0.0)	0 (0.0)	
Weakly positive	0 (0.0)	0 (0.0)	
Positive	0 (0.0)	3 (3.3)	
Strongly positive	20 (100.0)	88 (96.7)	
ASA	1 (5.0)	9 (9.9)	0.795
ASD	0 (0.0)	1 (1.1)	1.000

Data are n (%) or mean ± standard deviation; RLS, right-to-left shunt; ASA, atrial septal aneurysm; ASD, atrial septal defect; First syncope episode, the patient had only one episode of syncope and then came to the hospital.

The median length of stay in the hospital for the patients who underwent a procedure for PFO closure was 4 days. Postoperative pericardial tamponade was the only complication that occurred in the non-recurrent group, and no complications were found in the recurrent group. After a long follow-up (31.11 ± 14.30 months), 10 out of the 91 patients in the closure group had recurrent syncope, while 7 out of the 20 patients in the control group had recurrent syncope. The difference in the recurrent syncope rate between the two groups was statistically significant (11.0 vs. 35.0%, *P* < 0.05), as shown in Table 2.

**Table 2:** Comparisons of syncope recurrence between the closure group and the control group.

	**Control group** **(*n* = 20)**	**Closure group** **(*n* = 91)**	***P*-value**
Recurrence of syncope	7 (35.0)	10 (11.0)	0.018
Follow-up period (months)	32.65 ± 14.20	30.77 ± 14.36	0.594

Data are n (%) or mean ± standard deviation.

To further explore the factors that influence the PFO closure outcome of patients with unexplained syncope, we divided the closure group into the non-recurrent group (*n* = 81) and the recurrent group (*n* = 10). The baseline data and ultrasound characteristics of the patients in the two groups are compared in Table 3. The recurrent group was less likely to have combined triggers (10 vs. 59.3%, *P* = 0.009), more likely to have hypertension (30 vs. 6.2%, *P* = 0.041), and had more syncope episodes (> five times) (60 vs. 23.5%, *P* = 0.039) than the non-recurrent group. There were no significant differences in sex, age, history of syncope, seizure time, PFO-associated brain lesions, migraine, dizziness, epilepsy, diabetes mellitus, or baseline ultrasound characteristics between the two groups.

**Table 3:** Comparisons of baseline data and ultrasound characteristics between the non-recurrent group and the recurrent group.

	**Non-recurrent group** **(*n* = 81)**	**Recurrent group** **(*n* = 10)**	***P*-value**
Sex: Women	51 (63.0)	6 (60.0)	1.000
Age (years)	41.05 ± 15.26	37.50 ± 17.90	0.547
Time of syncope history			0.131
Several days	10 (12.3)	0 (0.0)	
Several months	25 (30.9)	2 (20.0)	
Several years	46 (56.8)	8 (80.0)	
Duration of syncope onset			0.431
Several seconds	23 (28.4)	1 (10.0)	
Several minutes	47 (58.0)	8 (80.0)	
≥10 min	11 (13.6)	1 (10.0)	
Number of syncope episodes			0.009
1 time	21 (25.9)	0 (0.0)	
2–5 times	41 (50.6)	4 (40.0)	
>5 times	19 (23.5)	6 (60.0)	
First syncope episode	19 (23.5)	0 (0.0)	0.190
Syncope symptoms alone	29 (35.8)	7 (70.0)	0.081
Syncope triggers	48 (59.3)	1 (10.0)	0.009
PFO-associated brain lesions	7 (8.6)	0 (0.0)	1.000
Migraine	27 (33.3)	2 (20.0)	0.621
Dizziness	20 (24.7)	1 (10.0)	0.521
Epilepsy	4 (4.9)	0 (0.0)	1.000
Hypertension	5 (6.2)	3 (30.0)	0.041
Diabetes mellitus	1 (1.2)	1 (10.0)	0.209
Size of atrial thin zone (mm)	17.60 ± 4.84	18.90 ± 3.35	0.258
Preoperative resting RLS grade			0.335
No	52 (64.2)	4 (40.0)	
Small	13 (16.0)	5 (50.0)	
Middle	3 (3.7)	0 (0.0)	
Large	13 (16.0)	1 (10.0)	
Preoperative right echocardiography			1.000
Positive	3 (3.7)	0 (0.0)	
Strongly positive	78 (96.3)	10 (100.0)	
ASA	8 (9.9)	1 (10.0)	1.000
ASD	1 (1.2)	0 (0.0)	1.000

Data are n (%) or mean ± standard deviation; RLS, right-to-left shunt; ASA, atrial septal aneurysm; ASD, atrial septal defect; First syncope episode, the patient had only one episode of syncope and then came to the hospital.

The details of the operation and postoperative follow-up of patients in the two groups are compared in Table 4. In the non-recurrence group, 78 patients were treated with the Cari-O-Fix PFO occluder, among whom PF1825 was used in 66 patients, PF3030 was used in 2 patients, PF2535 was used in 10 patients, and ASDO was used in 3 patients. In the recurrence group, the Cari-O-Fix PF1825 occluder was used for all patients. The difference between the two groups in the type of occluder used was not statistically significant (*P* > 0.05). The effective closure rates in the non-recurrence group at 1, 6, and 12 months after closure were 79.0, 91.4, and 91.4%, respectively. The effective closure rate in the recurrence group was 60.0%, unchanged at 1, 6, and 12 months after closure. There was a significant difference in the effective closure rate between 6 and 12 months after closure (*P* < 0.05). For the residual RLS, there was no significant difference between the two groups at 1 and 6 months after closure (*P* > 0.05), while the difference at 12 months after closure was statistically significant (*P* < 0.05).

**Table 4:** Comparisons of operation and follow-up between the non-recurrent group and the recurrent group.

	**Non-recurrent group** **(*n* = 81)**	**Recurrent group** **(*n* = 10)**	***P*-value**
Type of occluder			1.000
Cari-O-fix PFO	78 (96.3)	10 (100.0)	
PF1825	66 (81.5)	10 (100.0)	
PF3030	2 (2.5)	0 (0.0)	
PF2535	10 (12.3)	0 (0.0)	
ASDO	3 (3.7)	0 (0.0)	
Complications	1 (1.2)	0 (0.0)	1.000
Residual RLS at 1 month after closure			0.404
No	45 (55.6)	5 (50.0)	
Small	19 (23.5)	1 (10.0)	
Middle	8 (9.9)	1 (10.0)	
Large	9 (11.1)	3 (30.0)	
Effective closure at 1 month after closure	64 (79.0)	6 (60.0)	0.343
Residual RLS at 6 months after closure			0.050
No	59 (72.8)	5 (50.0)	
Small	15 (18.5)	1 (10.0)	
Middle	6 (7.4)	2 (20.0)	
Large	1 (1.2)	2 (20.0)	
Effective closure at 6 months after closure	74 (91.4)	6 (60.0)	0.018
Residual RLS at 12 months after closure			0.040
No	61 (75.3)	5 (50.0)	
Small	13 (16.0)	1 (10.0)	
Middle	6 (7.4)	3 (30.0)	
Large	1 (1.2)	1 (10.0)	
Effective closure at 12 months after closure	74 (91.4)	6 (60.0)	0.018
Length of stay in hospital (days)	4 (3.0,5.0)	3.5 (2.8,6.0)	0.674

Data are n (%) or mean ± standard deviation; PFO, patent foramen ovale; RLS, right-to-left shunt.

In the univariate logistic regression analyses comparing the non-recurrence group with the recurrence group after PFO closure, fewer preoperative syncope triggers, more preoperative syncopal episodes, hypertension, a low effective closure rate at 6 and 12 months after closure, and postoperative 12 months of residual RLS were significantly correlated with more recurrence (Table 5). After removing the intermediate variables, the possible significant factors in the univariate logistic regression analysis (*P* < 0.05) were included in the multifactorial logistic regression analysis (Table 6). Multifactorial logistic regression analysis confirmed that the number of syncope episodes (OR, 5.15; 95% CI, 1.03–25.73; *P* < 0.05), syncope triggers (OR, 0.03; 95% CI, 0.00–0.52; *P* < 0.05), and hypertension (OR, 18.79; 95% CI, 1.30–271.63, *P* < 0.05) were independent prognostic factors that predicted the syncope outcome after PFO closure. In addition, residual RLS 12 months after closure (OR, 2.62; 95% CI, 1.06–6.45; *P* < 0.05) was associated with syncope recurrence after PFO closure.

**Table 5:** Factors influencing syncope recurrence after PFO closure (univariate logistic regression).

**Factors**	**Group**	***P*-value**	**OR (95% CI)**
Number of syncope episodes		0.014	4.37 (1.34–14.27)
Syncope triggers	No^*^		
	Yes	0.017	0.08 (0.01–0.63)
Hypertension	No^*^		
	Yes	0.024	6.51 (1.28–33.16)
Effective closure at 6 months after closure	No^*^		
	Yes	0.010	0.14 (0.03–0.63)
Effective closure at 12 months after closure	No^*^		
	Yes	0.010	0.14 (0.03–0.63)
Residual RLS at 12 months after closure		0.018	2.33 (1.16–4.71)

* : control group; PFO, patent foramen ovale; RLS, right-to-left shunt.

**Table 6:** Factors influencing syncope recurrence after PFO closure (multifactorial logistic regression).

**Factors**	**Group**	***P*-value**	**OR (95% CI)**
Number of syncope episodes		0.046	5.15 (1.03–25.73)
Syncope triggers	No^*^		
	Yes	0.015	0.03 (0.00–0.52)
Hypertension	No^*^		
	Yes	0.031	18.79 (1.30–271.63)
Residual RLS 12 months after closure		0.037	2.62 (1.06–6.45)

* : control group; PFO, patent foramen ovale; RLS, right-to-left shunt.

## 4. Discussion

In the present study, after a long-term follow-up, we found that the rate of recurrent syncope in the closure group decreased by 24.0% compared to that in the control group. In addition, the difference was statistically significant. It is well-known that syncope is a common disease with a difficult etiology. According to the European guidelines on syncope, it is estimated that 40% of the population will experience syncope at least once in their lifetime (21). Data from the Framingham study showed that the annual incidence of syncope was 6.2 cases per 1,000 people and that there were 500,000 new cases of syncope and 170,000 recurrent episodes of syncope each year (23, 24). The etiology still cannot be determined in approximately 50% of patients after a comprehensive neurological and cardiovascular examination (25). As a result of their high incidence and recurrence rate, repeated syncope attacks can cause disability and death, affecting both patients' quality of life and physical health. Therefore, identifying risk factors or predisposing factors in patients with unexplained syncope is crucial for effective preventive measures. In 2000, Karinc et al. (26) found that 50% of patients with a PFO-related stroke had a history of syncope or palpitations. In 2016, a study (27) found that the incidence of PFO in patients with unexplained syncope was 75.4%, which was significantly higher than the detection rate of PFO in the general population (25%). In this study, 2 out of 26 patients who underwent PFO closure had a recurrence of syncope (7.6%), while 5 out of 20 patients who did not undergo surgery had a recurrence of syncope (25%). The recurrence rate was significantly higher than that of patients who underwent an operation, but the difference was not statistically significant. The short follow-up time, the small number of cases and the fact that PFOs were not all large shunts are limitations of this study. In a recent study (28), it was found that the incidence of RLS in patients with unexplained syncope was significantly higher than that in normal controls, and most of the right-to-left shunting was through PFO (29). Therefore, considering these results along with the results of previous studies, we conclude that PFO is highly correlated with unexplained syncope and that PFO closure can prevent the recurrence of syncope. However, we found significant differences between the closure group and the control group in terms of first syncope episode, syncope triggers, syncope symptoms only, and combined headache in the comparison of baseline data. In the control group, the number of first syncope episodes and the number of syncope symptoms alone were higher, while the number of combined triggers and the number of combined headache symptoms were lower. Overall, patients in the control group who chose conservative treatment had milder symptoms. Even though these baseline differences may have caused bias in the results, we still concluded that the difference in the recurrence rate of syncope between the two groups was statistically significant, indicating that the difference in the recurrence rate of syncope between the two groups might be greater if the symptoms were the same.

Our prognostic factor analysis found that syncope triggers increased the likelihood of a favorable outcome for syncope prevention. These syncope triggers can cause changes in abdominal pressure, thus causing a transient increase in right atrial pressure. Normally, the pressure of the left atrium is 3–5 mmHg higher than that of the right atrium. Thus, the foramen ovale closes without causing blood diversion. However, when the right atrial pressure increases, such as during Valsalva action, sneezing, violent coughing, laughing, and childbirth, the sudden increase in the right atrial pressure will push open the weak primary septum to the left side. The venous blood from the right atrium will flow into the left atrium, forming an RLS. Therefore, as the heart pumps blood to the brain and other organs, thrombi, air thrombi, and vasoactive substances can lead to conditions such as cerebrovascular events in a process known as paradoxical embolism (30). In recent years, many studies (31, 32), including large clinical trials, have found that PFO is closely related to ischemic stroke and migraine. The mechanism is likely to be related to paradoxical embolism. Mo Li et al. (27) found that patients with PFO who had a large right-to-left shunt were more likely to experience syncope during exercise or changes in abdominal pressure. The main mechanism of syncope in PFO patients may be paradoxical embolism. Therefore, we speculate that the possible mechanism of syncope caused by PFO is as follows: (1) The emboli formed by the repeated opening of the foramen ovale or by lower extremity veins fall off and enter the left atrium, from which they are pushed into the systemic circulation with the pumping of blood, resulting in arterial embolisms that may cause stroke, myocardial infarction, and syncope (33, 34). (2) Venous blood is largely shunted from the right atrium to the left heart system and mixed arteriovenous blood supplies the brain, which can cause transient cerebral ischemia and hypoxia, resulting in syncope (35–37). At the 2022 TCT conference, Muhammed Rahim gave a presentation titled “Recurrent Syncope and Hypoxia Causing Platypnea-Orthodeoxia Syndrome Due to a Patent Foramen Ovale.” (3) Due to extreme RLS activity in the heart, vasoactive substances (such as 5-hydroxytryptamine, etc.) from the venous circulation enter the left heart system. They are pumped to the brain in the blood, causing cerebral artery spasms and resulting in a transient blood shortage in the brain.

In our data, the effective closure rate 12 months after closure was significantly different between the two groups. At present, the efficacy and safety of PFO closure have been widely recognized, but the incidence of residual shunting after closure is still high; as many as 25% of patients have a residual shunt, and nearly 10% of patients have moderate-to-large amounts of residual shunting (6, 38). Moreover, residual shunting also indicates poor effectiveness of PFO closure, which is closely related to the increased risk of stroke recurrence (39) and the low degree of improvement of migraine symptoms (40). Therefore, the low effective closure rate in the recurrence group may be a reason for syncope recurrence. It also reinforces the strong relationship between PFO and unexplained syncope.

The following factors limited the present study. First, our study was a retrospective and single-center design, which may lead to neglect and underestimation of some significant predictors. Second, the small sample size of the control group may magnify or diminish the effect of PFO closure. Third, the follow-up time was 11–62 months, which may not be long enough to observe syncope recurrence. Finally, there is little evidence that PFO occlusion can effectively prevent the recurrence of unexplained syncope.

## 5. Conclusions

In our study, PFO closure reduced the recurrence of unexplained syncope. Prevention efficacy was prognosticated by factors including the presence or absence of syncope induction, the frequency of syncope episodes, and the presence or absence of hypertension. The recurrence of syncope was also related to residual shunts post closure. However, this was a small retrospective study and does not provide definitive proof that PFO closure effectively reduced syncope recurrence. In the future, we will expand our scope based on the current indication through dedicated clinical trials.

## Data availability statement

The raw data supporting the conclusions of this article will be made available by the authors, without undue reservation.

## Ethics statement

The studies involving human participants were reviewed and approved by the Research Ethics Committee of the Xiangya Hospital. Written informed consent to participate in this study was provided by the participants' legal guardian/next of kin.

## Author contributions

XW reviewed the literature and edited the manuscript. XL and LZ substantially revised the manuscript for readability and intellectual content. YL, ZG, and JD contributed to the table design and manuscript revision. XC contributed expert medical advice and manuscript revision. All authors read and approved the final submitted version.
